# Targeting the IGF-Axis for Cancer Therapy: Development and Validation of an IGF-Trap as a Potential Drug

**DOI:** 10.3390/cells9051098

**Published:** 2020-04-29

**Authors:** Yinhsuan Michely Chen, Shu Qi, Stephanie Perrino, Masakazu Hashimoto, Pnina Brodt

**Affiliations:** 1 Department of Medicine, Division of Experimental Medicine, McGill University, Montreal, QC H3A 0G4, Canada; 2 The Research Institute of the McGill University Health Center, Montreal, QC H4A 3J1, Canada; 3 Department of Surgery, McGill University, Montreal, QC H3A 0G4, Canada; 4 Department of Oncology, McGill University, Montreal, QC H3A 0G4, Canada

**Keywords:** IGF-I receptor, signaling, targeted therapeutics, IGF-Trap

## Abstract

The insulin-like growth factor (IGF)-axis was implicated in cancer progression and identified as a clinically important therapeutic target. Several IGF-I receptor (IGF-IR) targeting drugs including humanized monoclonal antibodies have advanced to phase II/III clinical trials, but to date, have not progressed to clinical use, due, at least in part, to interference with insulin receptor signaling and compensatory signaling by the insulin receptor (IR) isoform A that can bind IGF-II and initiate mitogenic signaling. Here we briefly review the current state of IGF-targeting biologicals, discuss some factors that may be responsible for their poor performance in the clinic and outline the stepwise bioengineering and validation of an IGF-Trap—a novel anti-cancer therapeutic that could bypass these limitations. The IGF-Trap is a heterotetramer, consisting of the entire extracellular domain of the IGF-IR fused to the Fc portion of human IgG_1_. It binds human IGF-I and IGF-II with a three-log higher affinity than insulin and could inhibit IGF-IR driven cellular functions such as survival, proliferation and invasion in multiple carcinoma cell models in vitro. In vivo, the IGF-Trap has favorable pharmacokinetic properties and could markedly reduce metastatic outgrowth of colon and lung carcinoma cells in the liver, outperforming IGF-IR and ligand-binding monoclonal antibodies. Moreover, IGF-Trap dose-response profiles correlate with their bio-availability profiles, as measured by the IGF kinase receptor-activation (KIRA) assay, providing a novel, surrogate biomarker for drug efficacy. Our studies identify the IGF-Trap as a potent, safe, anti-cancer therapeutic that could overcome some of the obstacles encountered by IGF-targeting biologicals that have already been evaluated in clinical settings.

## 1. Background Information

### 1.1. The Insulin-Like Growth Factor (IGF)-Axis

The IGF-axis consists of two cell surface receptors (IGF-IR and IGF-IIR), the ligands IGF-I and IGF-II, high affinity binding proteins (IGFBP-1-6) and their proteases (reviewed in [1,2]. IGF-IR shares a 60% sequence homology with the insulin receptor (IR). It is synthesized as a polypeptide precursor that undergoes post translational modification (glycosylation, proteolytic cleavage and dimerization) to form a heterotetramer composed of two α and two β subunits linked by α–α and α–β disulphide bonds. The α subunits are extracellular and contain the ligand binding site, while the β subunits have an extracellular domain, a transmembrane domain and an intracellular portion that contains the tyrosine kinase domain [3]. 

Upon ligand binding, the tyrosine kinase domain in the β subunit is activated, inducing a conformational change that leads to autophosphorylation at Tyr950 that serves as a docking site for signalling substrates including the insulin receptor substrate (IRS) proteins IRS-1-4, and the activation of PI3-kinase (PI3K)/Akt/ mammalian target of rapamycin (mTOR) and Raf /MEK/ERK signaling. This leads to regulation of cell survival and protein synthesis on one hand, and gene expression, cellular proliferation and differentiation, on the other [4,5].

IGF-IR can also translocate to the nucleus in a ligand-dependent manner following SUMOlyation of three lysine residues on the β-subunit [6]. In the nucleus, IGF-IR can act as a transcriptional co-activator with LEF/TCF, increasing promoter activity of the downstream target genes cyclin D1 and Axin2, upregulating their expression and promoting cell cycle progression [7]. In several human malignancies including clear cell renal cancer, colorectal carcinoma and pediatric glioma, nuclear IGF-IR was associated with advanced disease and adverse prognosis [8,9,10]. Codony-Servat el al. showed that in colorectal carcinoma cells treated with IGF-IR-blocking antibodies, nuclear translocation increased, suggesting that nuclear sequestration of the receptor could contribute to therapy resistance [9]. 

### 1.2. Hybrid Receptors and Crosstalk with Other Receptors

Two IR isoforms, IR-A and IR-B, formed by the alternative splicing of exon 11, have been identified [11]. IR-A is expressed predominantly in embryonic and fetal tissues, in the central nervous system (CNS) and hematopoietic cells and is frequently upregulated in cancer cells, whereas IR-B is expressed mainly in the liver, fat and muscle where it binds insulin with high affinity and mediates its metabolic functions. IR-A can bind IGF-II and insulin with high affinities, and this can initiate mitogenic signaling and tumorigenesis. RNA sequencing data based on analysis of 6943 samples, representing 21 tumor types in the Cancer Genome Atlas, revealed IR-A expression in all tumor types analyzed, and IR-B expression was also detected in many tumor samples. However the IR-A/IR-B ratio is generally in favor of the IR-A isoform in many cancer types including breast, colon, and lung carcinomas (extensively reviewed in [11,12].

Since many cancer cells overexpress both the insulin and IGF-I receptors and due to the high sequence homology between these receptors, hybrid receptors consisting of one insulin αβ hemi-receptor and one IGF-IR αβ hemi-receptor can also form. The IR-A/IGF-IR hybrids bind insulin and both IGF-I and IGF-II with similar high affinities, while IR-B/IGF-IR hybrids bind IGF-I with high affinity, IGF-II with lower affinity and insulin with poor affinity [13]. The specific signaling and functions of the hybrid receptors remain largely unknown, as they can bind and be activated by all three ligands. In a study of human breast carcinoma specimens and cell lines, hybrid receptor levels exceeded those of IGF-IR in a large proportion of specimens and in cultured cells; hybrid receptor autophosphorylation in response to IGF-I exceeded IGF-IR autophosphorylation and could initiate growth signaling, suggesting that these receptors could contribute to ligand mediated signal transduction [14] (reviewed in [15]). In a recent study using inducible chimeric receptors in mammary carcinoma cells, both IGF-IR and the hybrid receptor were found to induce cell proliferation, but only IGF-IR had anti-apoptotic effects [16], suggesting that it activates distinct signaling pathways. The high expression of IR-A in many cancer types and its ability to initiate mitogenic signaling in response to IGF-II, as well as the presence of signaling-competent hybrid receptors may have been a major factor in the outcome of clinical trials for IGF-IR targeting antibodies and other inhibitors and has emerged as a major consideration in the design of IGF-axis targeting drugs. 

Furthermore, IGF-IR/IR signaling is part of a complex network of receptor tyrosine kinase (RTK)-initiated pathways. The IGF-IR crosstalks with several RTKs including the epidermal growth factor receptor (EGFR), fibroblast growth factor receptor (FGFR), platelet-derived growth factor receptor (PDGFR) and human epidermal growth factor receptor 2 (HER-2), as well as with the steroid hormone receptors, estrogen receptor (ER) and androgen receptor. For example, crosstalk between ERα and IGF-IR was demonstrated in uterine cells, where IGF-IR signaling could be triggered by estradiol/ER-mediated induction of IGF-I synthesis in vitro. Conversely, ER transcriptional activity could be induced by IGF-I, in an estradiol-independent manner and IGF-I-induced ER transcriptional activity could be induced in the uteri of ovariectomized mice in vivo [17,18]. Crosstalk between IGF-IR and EGFR and compensatory actions between their signaling pathways have been identified as potential resistance mechanisms to drugs that target either of these axes. Thus, treatment of head and neck squamous cell carcinoma and non-small cell lung cancer cells with the fully humanized anti-IGF-IR monoclonal antibody (MAb), Cixutumumab, induced Akt and mammalian target of rapamycin (mTOR) activation, resulting in EGFR, Akt1, and survivin synthesis and EGFR pathway activation. This inter-dependence and compensatory RTK signaling has been identified as another obstacle to successful therapeutic targeting of the IGF-axis. 

### 1.3. Targeting of the IGF-Axis for Cancer Therapy—The Rational

Increased expression of IGF-IR and/or its ligands has been documented in many human malignancies such as lung, breast, colon and prostate carcinoma, glioblastoma and melanoma, and high expression levels were shown to be associated with metastasis, shorter survival and poor prognosis [19], identifying this axis as a target for cancer therapy. High circulating IGF-I levels were identified as a predictive factor in several malignancies including lung, breast, colorectal and prostate carcinoma [20], and thought to contribute to cellular transformation and malignant progression [2]. The important role of circulating IGF-I in cancer development was demonstrated in vivo using liver-specific-IGF-I deficient (LID and iLID) mouse models where decreased mammary tumor incidence and progression and reduced colon adenocarcinoma growth and metastasis were documented [21,22]. This was also observed in other mouse models of reduced circulating IGF-I levels such as the lit/lit mice that have only 10% of normal circulating IGF-I levels due to reduced growth hormone (GH) production and in dw/dw dwarf mice that are deficient in GH and IGF-I production [22,23]. In addition to circulating IGF-I, tissue IGF-I levels that activate IGF-IR signaling in a paracrine or autocrine fashion were also shown to contribute to tumorigenesis in both animal models and human studies [24,25,26]. In a study of 125 primary non-small cell lung cancer compared to benign pulmonary lesions, high IGF-I and IGF-IR levels were associated with advanced-stage disease and expression of IGF-I correlated with tumor size and poor outcome [27]. However, in contrast to these findings, tumor IGF-I levels were found to be associated with better overall survival in studies of prostate and breast cancer tumors [28,29]. This may reflect the dual role of IGF-I as a proliferation and differentiation factor, depending on the cellular context [29,30,31,32]. As these studies were based on immunohistochemical evaluation or gene expression analyses performed on whole tumor tissue, the precise source of IGF-I in these studies cannot be definitively identified. The relative contributions of circulating and local IGF-I levels to malignant progression and the role of IGFBPs in modulating their effects remain an open question with implications for IGF-targeting and patient stratification [33].

The IGF ligands form complexes with six high-affinity IGFBPs that modulate their half-life and bioavailability [34]. Lower circulating IGFBP levels were found to be associated with increased risk for several cancers including premenopausal breast carcinoma, prostate carcinoma, colorectal carcinoma, lung cancer, endometrial cancer and bladder cancer [2]. The identification of all components of the axis as contributors to the development of malignant disease has spurred an intensive effort to design inhibitors and strategies for blockade of IGF-IR signaling. These inhibitors can be broadly divided into drugs that target the receptor (monoclonal antibodies and small molecule tyrosine kinase inhibitors (TKI)) and strategies that reduce ligand bioavailability to the cognate receptor. A brief summary of the experience with these drugs is provided below and in Table 1.

#### 1.3.1. IGF-Targeting for Cancer Management: The Current Landscape and Overall Clinical Experience

##### Targeting the IGF-I Receptor

Receptor-specific antibodies: IGF-IR antibodies can inhibit signaling by binding to the extracellular α subunits, blocking ligand binding and triggering receptor internalization. Several humanized or fully human neutralizing anti-IGF-IR antibodies have entered clinical trials. Included among them are cixutumumab (IMC-A12-ImClone, New York, NY, USA), Figitumumab (CP-751,871-Pfizer, New York, NY, USA), Dalotuzumab (MK-0646; h7C10-Pierre Fabre (Paris, France) and Merck (Kenilworth, NJ, USA)), ganitumab (AMG 479-Amgen Thousand Oaks, CA, USA), Teprotumumab (R1507-Genmab (Copenhagen, Denmark) and Roche (Basel, Switzerland)), Robatumumab (SCH 717454, 19D12-ImmunoGen (Waltham, MA, USA) and Sanofi (Paris, France)), Istiratumab (MM141-Merrimack (Cambridge, MA, USA)), BIIB022 (Biogen (Cambridge, MA, USA)), and AVE1642 (EM164-Biogen, Cambridge, MA, USA). Unfortunately, the use of most of these drugs in cancer therapy has been discontinued after several obstacles were identified [43]. IGF-IR blocking drugs could cause insulin resistance, hyperinsulinemia and mild hyperglycemia [43]. In addition, the therapeutic responses to the monoclonal antibodies were disappointing, and this was attributed to several potential factors including: (i) a compensatory feedback mechanism that leads to increased IGF production due to increased growth hormone release [44], (ii) IR-A signalling that can be initiated by IGF-II (the main plasma IGF-IR ligand in human) and leads to mitogenic signaling; and (iii) cancer cell resistance due to activation of compensatory RTK signaling [36,45,46]. Several of the anti-IGF-IR antibodies have also been tested in combination with chemotherapy or antibodies to other RTKs [47,48]. Despite pre-clinical data to suggest that these combinations could be effective in targeting resistant tumor subpopulations [49,50,51,52,53,54,55,56], the results of clinical trials have generally been disappointing, resulting in termination due to lack of demonstrable efficacy [43,57]. An exception may be teprotumumab that had a successful phase III clinical trial with thyroid eye diseases and has been U.S. Food and Drug Administration (FDA)-approved for the treatment of Graves’ disease [58,59]. To address potential resistance due to activation of other RTKs, bispecific antibodies that target a second kinase have been generated. These include XGFR, a bispecific anti-IGF-IR/EGFR antibody that showed inhibition of tumor growth and enhanced immune activation in pancreatic cancer in vivo [39], and Istiratumab (MM-141) which co-targets IGF-IR and ErbB3. MM-141 was tested in combination with standard of care (SOC) chemotherapy in a phase II clinical trial for pancreatic cancer, but failed to show a survival advantage in comparison to SOC alone [60,61]. Of importance, however, are the reports that specific IGF-targeting drugs were generally well tolerated. 

Several small molecule tyrosine kinase inhibitors were also developed to target IGF-IR signaling including Masoprocol (INSM-18, NDGA – InsMed (Bridgewater Township, NJ, USA)), Linsitinib (OSI-906 – OSI (Farmingdale, NY, USA)), BMS-754807 (BMS (Montreal, QC, Canada)), AXL1717 (Picropodophyllin- Axelar AB (Solna, Sweden)) and XL-228 (Exelixis (Alameda, CA, USA)). A potential advantage of small TKIs is that they may also inhibit IR-A-initiated signaling due to the high homology between these receptors. However, this is a double-edged sword, as disruption of IR signaling can have deleterious effects on glucose metabolism and lead to hyperinsulinemia and hyperglycemia [36,37]. To date, no IGF-IR TKI has advanced to clinical use. 

##### Targeting the IGF-Ligands

An alternative approach to blocking IGF-IR signaling is targeting the ligands to reduce their bioavailability to the receptor. An advantage of this approach is that while it can inhibit IGF-IR and IR-A-derived mitogenic signaling, it has no direct effect on insulin-mediated metabolic functions. Two dual IGF-I/IGF-II neutralizing antibodies, Dusigtumab (MEDI-573-MedImmune, Gaithersburg, MD, USA) and Xentuzumab (BI-836845-Boehringer-Ingelheim, Ingelheim am Rhein, Germany), have entered phase I clinical trials [62,63] and had minimal adverse effects. However, the efficacy of ligand-neutralizing antibodies may be limited by cell surface expression levels of IGF-IR on the cancer cells, as they determine maximal ligand binding capacity [41].

IGFBPs are naturally occurring molecules that modulate the bioavailability of IGF ligands. IGFBP-3, the predominant IGFBP in the circulation can also induce IGF-independent apoptosis by mediating the pro-apoptotic function of TGFβ, in an IGF-IR independent manner. In addition, IGFBP-3 plays a role in the DNA repair response to DNA-damaging therapy and was shown to co-translocate to the nucleus of breast cancer cells with EGFR and DNA-dependent protein kinase in response to DNA damage, to mediate this function [64,65]. Recombinant human rhIGFBP-3 was shown to potentiate the effect of Herceptin on Herceptin-resistant human breast cancer cells in vitro as well as in a xenograft model in vivo by reducing Akt and ERK signaling [66] and an exogenously administered protease-resistant IGFBP-2 was shown to inhibit the growth of breast cancer cells in vitro and in a xenograft model in vivo [67]. However, to date, IGFBPs have not advanced to clinical testing, possibly because of their short half-life in vivo. 

## 2. Traps in the Clinic—Advantages and Challenges

An effective strategy for blocking the action of cell surface receptors is the use of soluble decoys that bind the ligand with high affinity, reducing its bioavailability to the cognate receptor in a highly specific manner [68,69,70]. The efficacy of such decoys can be significantly improved by the addition of an IgG Fc domain resulting in a more stable ligand known as “Trap”. For example, a soluble tumor necrosis factor (TNF)-α receptor-Fc fusion protein (Etanercept, Enbrel^®,^ approved in 1998) is currently in routine clinical use for the treatment of inflammatory conditions such as rheumatoid arthritis [71]; Interleukin (IL)-1-Trap (Rilonacept, Arcalyst^®^, approved in 2008) is used for the treatment of cryopyrin-associated periodic syndromes (CAPS) [72], and a VEGFR1/VEGFR2-Fc decoy (VEGF-TRAP-Aflibercept, Regeneron (Eastview, NY, USA)) was approved for the treatment of wet macular degeneration under the trade name Eylea and for metastatic colorectal cancer as Zaltrap [68]. Although the development of IGF-IR decoys for cancer treatment has been reported [73], to date, none have advanced into clinical use.

To construct high affinity and high efficacy ligand binding Traps, two or more distinct receptor domains have to be fused to the Fc molecule. This fusion strategy can result in highly potent therapeutic drugs. For example, Rilonacept was engineered with the extracellular domains of the IL-1 receptor (IL-1R1) and the IL-1R accessory protein (IL-1-RAcP) fused to the Fc domain of human IgG_1,_ resulting in a potent (IC_50_ = 6.5 pM), high affinity (Kd = 1.5 pM) IL-1R antagonist [72,74]. Aflibercept is composed of the ligand-binding domains of VEGFR1 and VEGFR2 fused to the Fc domain of IgG_1_ and has a higher affinity to multiple isoforms of VEGF than the VEGF targeting MAbs. It was consequently found to be more effective than the MAbs Ranibizumab and Bevacizumab in patients with marked loss of visual acuity [75], and a superior inhibitor of angiogenesis in a model of neuroblastoma, where it caused regression of coopted vascular structures at high doses [76]. Recently, an Fc-fusion EGFR decoy comprising the truncated extracellular domains of EGFR/ErbB-1 and ErbB-4 fused to Fc was shown to have high-affinity ligand binding to EGF-like growth factors and could inhibit the invasive growth and metastasis of mammary carcinoma cells [77]. 

An advantage of Fc fusion proteins is their increased stability and extended half-life in vivo that is mediated primarily through their binding to the neonatal Fc receptor (FcRn) and their reduced renal elimination [78]. This increase in half-life reduces the dosing frequency and immunogenicity of the fusion proteins with clear clinical benefits. This was shown for both Etanercept [75,79] in the treatment of rheumatic diseases and for Aflibercept in the treatment of age-related macular degeneration [75]. Other advantages conferred by the Fc portion of Trap proteins, particularly in the context of cancer treatment, are their ability to trigger antibody-dependent cell-mediated cytotoxicity (ADCC) and antibody-dependent cell-mediated phagocytosis through Fc binding to Fc-gamma receptors (FcγRs) [80], and the activation of complement-dependent cytotoxicity (CDC) by the binding of complement C1q, leading to tumor cell killing [81,82]. 

The development of Fc-based therapeutics can be challenging. In order to improve recombinant protein expression, protein folding and protein stability, various modifications to the protein are required that can result in undesirable consequences such as altered protein–protein interactions, high molecular weight complex formation and aggregation, resulting in decreased bioactivity and increased risk of immunogenicity [83]. For example, removal of terminal carbohydrate moieties is an efficient way to eliminate undesired effector functions, but de-glycosylation can lead to instability and protein aggregation [84]. Moreover, even small changes in the amino acid sequence can have a considerable effect on the stability and safety profile of a drug [83]. The rapid emergence of technologies for protein engineering and modification will necessitate careful assessment of the risk/benefit profile, before they are transitioned to clinical use. 

## 3. The IGF-Trap—A Stepwise Bioengineering Venture

### 3.1. IGF-IR Decoys-Background Information

The identification of the IGF-axis as a target in cancer therapy has spurred many attempts to inhibit this axis through nucleic acid-based and protein-engineering strategies (Table 1). One early approach was the development, by several groups, of IGF-IR decoys that when secreted by the cancer cells, reduce ligand bioavailability to the cognate receptor and act as dominant negative receptor mutants. The Baserga group was first to report that transfection of a 486 amino acid (486/stop) truncated receptor into rat glioma C6 cells and subsequently, into human metastatic breast cancer MDA-MB-435 cells, resulted in the secretion of this receptor into the conditioned medium, inhibiting cancer cell invasion, increasing apoptosis and reducing colony formation in vitro. C6 cells expressing this decoy had reduced tumorigenesis in vivo, while MDA-MB-435 cells had reduced metastasis [73,85]. Sachdev et al. subsequently reported on the production of a C-terminal-truncated 262 bp IGF-IR decoy that retained the ligand binding domain but lacked the autophosphorylated tyrosine residues in the carboxyl terminus. They showed that LCC6 cells—a metastatic variant of breast carcinoma MDA-MB-435 cells—transfected with this truncated receptor lost their motility in response to IGF-I and the ability to metastasize in a xenograft model [86]. Min et al. analyzed the effects of two decoys of 482 and 950 amino acids in a xenograft model of human gastric cancer. Consistent with the above studies, they found that expression of these decoys suppressed tumorigenicity in vitro and in vivo, blocked ligand-induced Akt-1 activation and markedly increased the sensitivity of the cells to radiation and chemotherapy-induced apoptosis [87].

### 3.2. The Incremental Production/Validation Process for an IGF-Trap

**Figure 1:**
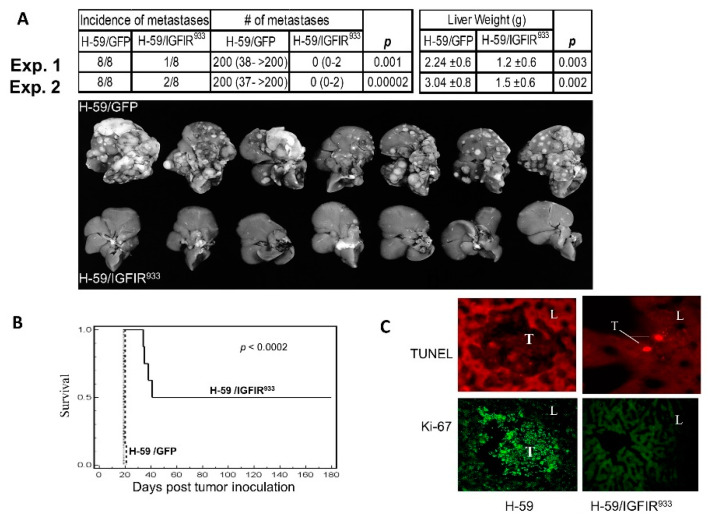
Loss of metastatic potential in lung carcinoma cells expressing a soluble IGF-IR decoy (IGFIR^933^). Lewis lung carcinoma subline H-59 cells were transduced with retroparticles expressing the truncated 933 aa IGF-IR decoy (H-59/IGFIR^933^) or GFP only (H-59/GFP) and 10^5^ tumor cells injected into syngeneic C57Bl/6 female mice via the intrasplenic/portal route to generate experimental liver metastases. Mice were sacrificed and visible metastases enumerated 14 days later. Shown in (**A**) (top) are the median numbers of metastases (and range) per liver based on eight animals per group in two separate experiments. Liver weights (means ± SD) are shown on the right, and representative livers from experiment (Exp.) 2 are shown on the bottom. Shown in (**B**) are survival data for mice inoculated in a similar manner (*p* < 0.0002) and in (**C**) terminal deoxynucleotidyl transferase (Tdt)-mediated nick end labeling (TUNEL) assay (top) and Ki-67 staining (bottom) performed on liver (L) cryostat sections prepared 5 days post tumor (T) injection (Mag. X135). Reproduced from [88].

**Figure 2:**
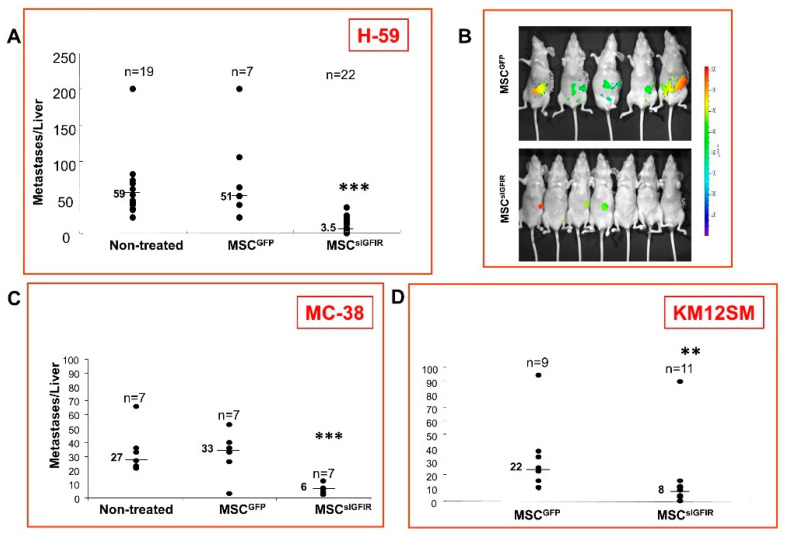
Bone marrow stromal cells producing a soluble IGF-IR inhibit experimental hepatic metastasis of lung and colon carcinoma. Syngeneic female C57Bl/6 (**A** and **C**) or nude (**B** and **D**) mice were implanted with 10^7^ genetically engineered marrow-derived stromal cells (MSCs) expressing sIGFIR (MSC^sIGFIR^) or control MSC (MSC^GFP^) embedded in Matrigel. Fourteen days later (**A**–**D**), the mice were inoculated via the intrasplenic/portal route with 10^5^ H-59 (**A** and **B**), 5 × 10^4^ murine colon carcinoma MC-38 (**C**) or 10^6^ human colon carcinoma KM12SM (**D**) cells. Mice were euthanized and liver metastases enumerated 14–16 (**A**), 18 (**C**) or 21 (**D**) days after or imaged using the IVIS 100 Xenogen 15 days (**B**) post tumor inoculation. Shown in (**A**) are the pooled data of three and in (**B**–**D**) individual experiments. Results of optical imaging are shown in (**B**). ** *p* < 0.01, *** *p* < 0.001, as determined by the non-parametric Mann–Whitney test. Reproduced from [89].

Our laboratory used a stepwise approach to engineer an IGF-Trap with potent growth inhibitory activity against multiple aggressive carcinomas. Initially a truncated (t) IGF-IR was engineered consisting of the first 933 amino acids and spanning the entire extracellular domain of the native receptor (IGF-IR^933^). This truncated receptor was expressed in highly metastatic murine lung carcinoma H-59 cells. We confirmed that these cells produced and secreted into the medium a (β^t^–α–α–β^t^) heterotetramer that neutralized exogenously added IGF-I and inhibited IGF-I-induced signaling and IGF-IR-mediated proliferation, invasion, and apoptosis resistance. Expression of this truncated receptor had a dramatic effect on the metastatic potential of H-59 cells, reducing hepatic metastases by 90% following their intrasplenic/portal inoculation and significantly extending the long-term, disease-free survival of the mice (Figure 1) [88]. These results identified the IGFIR^933^ as a potent anti-tumorigenic and anti-metastatic agent with potential applications for cancer therapy and prompted us to begin exploring the translational potential of this decoy as a biological therapeutic. Initially, two cell and gene therapy strategies were used. Namely, we genetically engineered autologous bone marrow stromal cells stably secreting the IGF-IR^933^ decoy and implanted them subcutaneously into mice to achieve sustained production of this decoy in vivo [89]. We confirmed that these cells were able to generate high plasma levels of sIGFIR for at least three weeks, with a longer duration in athymic nude mice, suggestive of immune-based elimination of the stromal cells in immunocompetent mice [89]. In mice implanted with IGF-IR^933^-producing stromal cells, a marked reduction in experimental hepatic metastases of colon and lung carcinoma cells was observed (Figure 2). Moreover, in hepatic micro-metastases, a significant reduction in intra-lesional angiogenesis and an increase in tumor cell apoptosis were seen, suggesting that the IGF-IR decoy impeded early events in the process of liver metastasis. The results showed that sustained delivery of a soluble IGF-IR decoy was highly effective in preventing the expansion of liver metastases. This was also confirmed when a second approach was used, namely when a gutless adenovirus expressing sIGFIR was injected into mice intravenously, leading to production of measurable sIGFIR plasma levels for up to 21 days and resulting in significant inhibition of experimental liver metastasis [90]. 

Having observed marked reductions in experimental liver metastases in mice with sustained high plasma levels of an IGF-IR decoy, and in an effort to expedite potential translation of this technology to the clinic, we used recombinant technology to engineer and scale-up production of an IGF-Trap with potent anti-cancer activity. This was achieved in a two-stage process. Initially, we generated the soluble receptor decoy expressed in CHO cells downstream of a cumate-inducible promoter, using lentivirus particles. CHO cell clones identified as high producers were expanded and protein production initiated by the addition of 1 mg/mL cumate followed by a 7–8-day incubation, before the soluble protein was harvested and a stepwise purification of sIGF-IR performed. High binding affinity of the recombinant protein for hIGF-I and a 10^3^-fold lower affinity for insulin were confirmed by surface plasmon resonance (SPR) and the biological activity of this protein was assessed and validated in multiple functional assays including IGF-initiated proliferation, invasion, anchorage independent growth and anoikis [91].

**Figure 3:**
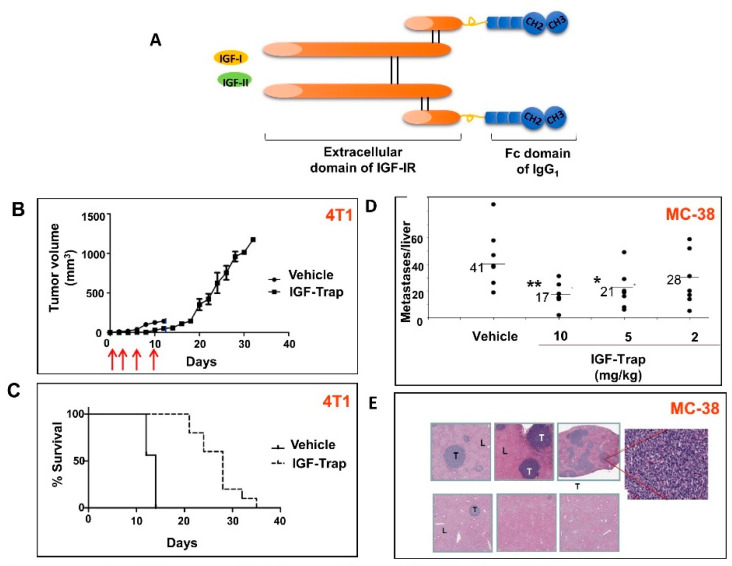
The IGF-Trap inhibits the orthotopic growth of mammary carcinoma and liver metastasis of colon carcinoma cells. Balb/c (**B** and **C**) or C57Bl/6 (**D** and **E**) mice were injected into the mammary fat pad (MFP) with 5 × 10^4^ 4T1 cells (**B** and **C**) or via the intrasplenic/portal route with 5 × 10^4^ MC-38 cells (**D** and **E**). IGF-Trap injections were administered i.v. to 4T1 injected mice 4 h and 3, 6 and 10 days (arrows) post tumor inoculation (10 mg/kg for the first 2 injections and 5 mg/kg subsequently) and to MC-38 injected mice, 24 h and 4 and 7 days post tumor inoculation. Shown in (**A**) is a diagrammatic representation of the 2^nd^ generation IGF-Trap. Shown in (**B**) are mean tumor volumes (±SD) and in (**C**) a Kaplan–Meier survival curve (*p* < 0.01 using Mantel-Cox or Gehan-Breslow-Wilcoxon Tests). Local MFP tumors grew rapidly in all untreated mice, causing morbidity by day 14, while in the treated mice, tumor growth was seen only after cessation of treatment. Shown in (**D**) are the numbers of visible liver metastases enumerated 18 days post tumor injection. Bars (and numbers) denote medians. Shown in (**E**) are representative hematoxylin and eosin-stained, formalin-fixed and paraffin-embedded sections obtained from different livers of MC-38-injected mice (magnification ×20; inset ×400). T: tumor; L: liver; * *p* < 0.05; ** *p* < 0.01. Reproduced from [91].

In order to improve the pharmacokinetic and potential therapeutic properties of this soluble receptor, thereby optimizing it for clinical translation, we then generated a sIGFIR–hFc–IgG_1_ fusion protein—the IGF-Trap—that was produced in CHO cells using a similar production/scale-up strategy (Figure 3). We found that the addition of the Fc fragment did not alter the individual binding kinetics or overall affinity of the recombinant protein. The IGF-Trap bound hIGF-I with highest affinity and hIGF-II and murine IGF-I with moderately lower affinities, and had a three-log weaker affinity for insulin, confirming the high affinity and specificity of the IGF-Trap and a binding profile consistent with that observed with the cognate cell surface receptor [91]. Similar to sIGFIR, the IGF-Trap inhibited IGF-IR signaling and IGF-I and IGF-II- regulated cellular functions in several carcinoma cell types including breast, lung and colon carcinoma cells in vitro. It had a favorable pharmacokinetic profile in vivo with a half-life of 47.5 h as compared to 21.9 h for sIGFIR, confirming that the addition of the two Fc domains improved the stability of this protein in vivo [91]. Moreover, IGF-Trap treatment inhibited the growth of human and murine breast carcinoma cells and markedly reduced experimental liver metastasis of colon and lung carcinoma in vivo (representative data shown in Figure 3). Interestingly, we found that the IGF-Trap had superior therapeutic efficacy to an anti-IGF-IR antibody or IGF-binding protein-1 when used at similar or higher concentrations in a human breast cancer model and experimental murine colon cancer metastasis assays, respectively.

### 3.3. A 3^rd^ Generation IGF-Trap—Properties, Bioactivity and Challenges

**Figure 4:**
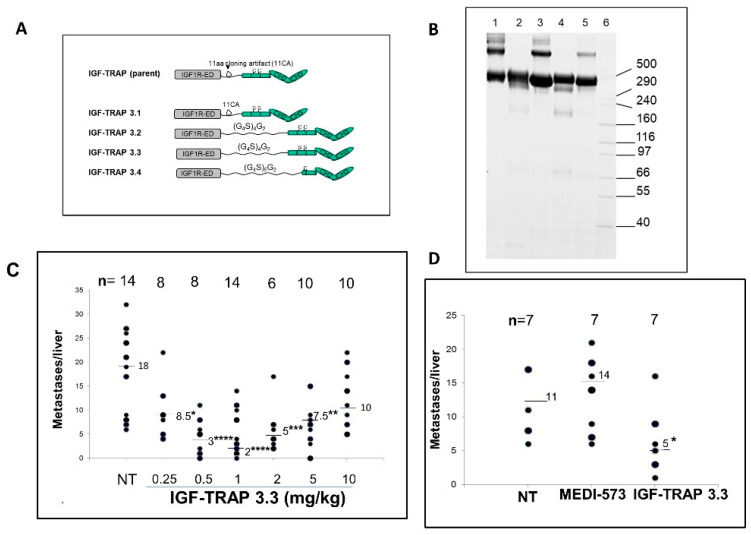
Cysteine-serine substitutions in the Fc domain of the IGF-Trap reduce high-molecular-weight (HMW) oligomers and improve pharmacodynamic properties. Shown in (**A**) is a schematic representation of the modifications engineered in the parent (2^nd^ generation) IGF-Trap and in (**B**) results of SDS-PAGE performed on purified parental or modified IGFIR-hFc-IgG_1_ proteins, using denaturing and non-reducing condition. Lanes: 1—parent IGF-Trap; 2—IGF-Trap 3.1; 3—IGF-Trap 3.2; 4—IGF-Trap 3.3; 5—IGF-Trap 3.4; 6—HMW protein standard (Invitrogen). Shown in (**C**) is the number of metastases enumerated in individual livers in three different experiments where mice were inoculated via the intrasplenic/portal route with 5 × 10^4^ MC-38 cells, treated with IGF-Trap 3.3 at the indicated doses from day 1 and thereafter twice weekly, for a total of 5 injections and sacrificed 16–18 days later. The total number of mice per treatment group is indicated on the top. Shown in (**D**) are results of a separate experiment where one group of mice was treated with 1 mg/kg of the anti-ligand MAb MEDI-573. Horizontal bars denote medians. NT: non-treated. * *p* < 0.05, ** *p* < 0.01, *** *p* < 0.005, **** *p* < 0.001, as assessed by the non-parametric Mann–Whitney test. Reproduced from [98].

A problem frequently encountered with Fc-fusion proteins is the formation of high-molecular-weight (HMW) complexes due to oligomerization by irregular disulfide bonding between adjacent Fc fragments [92,93]. The IGF-Trap is a tetramer with two β subunits, each fused to one Fc domain of IgG_1_, and this proximity of adjacent F_C_ domains lends itself to undesirable disulfide bonding and large complex formation. Indeed, we documented HMW protein species that migrated at the > 400 kDa range in the IGF-Trap preparations. We showed that these HMW species did not contribute significantly to the biologic activity of the Trap and could be minimized by step elution following Protein-A column purification [91]. In an effort to further improve the purity and manufacturability of the IGF-Trap, we therefore re-engineered the parent protein to eliminate such aberrant disulfide bonding by cysteine-to-serine substitutions in the hinge region of the human IgG_1_ Fc fragment, as well as by incorporating a longer and more flexible linker between the IGF-IR ectodomain and the Fc domain. Four different modified Traps were produced, and two were selected for further evaluation, based on a polyacrylamide gel profile that confirmed the elimination of HMW species in these preparations. We found that the IGF-Trap in which Cys-Ser substitutions in the Fc hinge region were combined with the addition of a flexible linker (IGF-Trap 3.3) had a considerably improved pharmacokinetic profile with a marked increase in the area under the serum concentration-time curve. Moreover, this IGF-Trap had an enhanced therapeutic profile, as evaluated in an experimental colon carcinoma liver metastasis model and was superior to a ligand binding antibody used under the same conditions (Figure 4). This indicated that depletion of HMW species and the increased stability also improved the pharmacodynamic properties of the Trap. 

The IGF kinase-receptor-activation (KIRA) assay measures ligand bioavailability by quantifying phosphorylated IGF-I receptor levels. While traditional end-point bioassays measure downstream effects of IGF-IR activation such as cell proliferation and survival, the KIRA assay is based on measuring receptor activation per se, thereby avoiding errors due to detection of other confounding signaling pathways. Moreover, naturally occurring IGFBPs and proteases in the circulation affect the bioavailability/bioactivity of IGF-I. While immune-based approaches such as enzyme linked immunoassays (ELISA) measure both total (BP-bound) and free ligand, the two-step KIRA assay provides a more accurate measure of bioactive ligands [94,95,96,97]. Using the KIRA assay, we found that IGF-I serum bioavailability correlated well with the IGF-Trap pharmacokinetic/pharmacodynamic profile, providing a novel, surrogate marker for its therapeutic efficacy [98]. 

## 4. Targeting the IGF-IR in the Tumor Microenvironment 

### 4.1. IGF-IR Is Expressed on Immune Cells and Plays a Role in Immunosuppression

The major immune cell subtypes (i.e., T and B lymphocytes), myeloid derived mononuclear cells and NK cells express the IGF-IR and are responsive to IGF ligands [99]. Although complex, there is compelling evidence that within a tumor microenvironment (TME), the IGF axis promotes an anti-inflammatory, immunosuppressive response that enables cancer expansion. Thus, IGF-I was shown to negatively regulate DC activation, impair their antigen-presenting function [100] and stimulate the proliferation of immunosuppressive regulatory T cells (Treg) [101,102]. IGF-IR activation was also linked to macrophage polarization to the pro-tumorigenic M2 phenotype [103,104]. Treatment of DC with the IGF inhibitor NVP-AEW541 restored DC-mediated antigen presentation and anti-tumor immunity [105]. A deficit in IGF-I signaling in macrophages was associated with a decreased capacity to induce the M2 state and an increased responsiveness to the pro-inflammatory cytokine IFNγ [104]. Moreover, the inhibitor NT157 that targets both the IGF-IR and STAT3 inhibited expression of pro-tumorigenic cytokines, chemokines and growth factors including IL-6, CCL2, CCL5 and TGFβ [106]. IGF-I was also shown to play a role in the survival of neutrophils by blocking Fas-mediated apoptosis [99]. Of interest, in patients treated with a MAb to IGF-IR (AMG 479), high levels of antibody binding to neutrophils were documented [107]. Finally, IGF-I may also play a role in the tumor-promoting effect of myeloid derived suppressor cells (MDSC) [108]. Collectively, these studies identify the IGF axis as a contributor to a pro-tumorigenic TME, suggesting that in addition to their direct positive effect on tumor cell survival and proliferation, the IGFs also potentiate escape mechanisms from immune-mediated tumor cell destruction.

### 4.2. Multiple Effects of the IGF-Trap on the Tumor Microenvironment

In addition to directly targeting IGF-signaling in the cancer cells, the IGF-Trap also had indirect effects on metastatic expansion by targeting the pro-metastatic microenvironment of the liver. As shown above, treatment with the IGF-Trap inhibited neovascularization in the early stages of metastases [91], suggesting that it affected endothelial cell migration and/or proliferation. Moreover, we have shown that IGF-I regulates hepatic stellate cell (HSC) activation in both cancer metastasis and cancer-free (CCl_4_-induced liver injury) models, and the IGF-Trap caused a significant reduction in HSC activation in response to metastatic colon cancer cells [109]. When analyzing neutrophil phenotypes in a colon cancer liver metastasis model, we also observed a reduction in CXCL4^high^/ICAM-1^low^ N2 polarized neutrophils in IGF-Trap treated mice that may potentially be mediated through regulation of TGFβ expression levels [110]. Finally, we have shown that IGF signaling regulates type IV collagen production in metastatic cancer cells, thereby promoting their growth in the liver [111,112]. Given the critical role that the extracellular matrix (ECM) plays in the TME [113], the IGF-Trap may therefore also impede metastatic expansion in this organ by altering the tumor-associated ECM. Thus, the IGF-Trap can have a multi-pronged effect on metastatic expansion, particularly in the liver, by impeding cancer cell proliferation, while also rendering the TME less hospitable to their expansion. 

### 4.3. Future Prospective: The Case for Combinatorial Therapy with the IGF-Trap

The TME in primary or secondary sites can either promote or suppress the progression of malignant disease. The nature of the immune response engendered within the TME is a major factor determining the balance between these opposing outcomes [114,115,116,117]. Recent advances in immunotherapy, based on targeting immune checkpoints such as PD-1 and CTLA-4 have yielded promising therapeutic results in several aggressive and treatment-refractory cancers such as malignant melanoma, small cell lung cancer and renal cell carcinoma [118,119,120]. To date, however, immunotherapy has failed to show promise in the treatment of malignancies such as colorectal carcinoma and pancreatic ductal adenocarcinoma that metastasize to the liver [121,122]. This may be due, at least in part, to the presence of immunosuppressive cells such MDSC and M2 macrophages that impede T cell mediated cytotoxicity. Thus, therapeutic approaches that can target an immunosuppressive TME and enhance the efficacy of immunotherapy are currently being sought [122,123]. As reviewed above, the major innate and adaptive immune cell subtypes express IGF-IR and are responsive to IGF ligands [99]. Although the role of IGF-IR in the development and function of immune cells is complex, there is compelling evidence that within the TME, the IGF axis promotes an anti-inflammatory, immunosuppressive response that enables cancer expansion. Thus, IGF targeting was shown to alter the tumor immune ME in colon cancer, reducing anti-inflammatory cytokines [106] and our own data identified IGF-IR on neutrophils and HSCs as a contributor to liver metastasis [109,110]. Collectively, these data provide a compelling rationale for combinatorial immunotherapy using immune checkpoint inhibitors together with IGF-targeting drugs. These combinations may be particularly effective for malignancies of the gastrointestinal track that metastasize to the liver, an organ with an innate immune hyporeactivity and the site of IGF-I production. Our bio-distribution studies have identified the liver as a major site for IGF-Trap accumulation, possibly due to the high local level of IGF-I [98]. This suggests that the IGF-Trap may be particularly well suited for combinatorial immunotherapy in liver-metastatic diseases. 

## 5. Conclusions

Clinical trials with IGF-targeting biologicals exposed several obstacles to their successful use in cancer therapy. Due to the homology and crosstalk between IGF-IR and IR, several inhibitors of IGF-IR signaling (including tyrosine kinase inhibitors) were found to also disrupt IR signaling, resulting in undesirable side effects such as hyperinsulinemia and hyperglycemia. The responses to more specific drugs, such as anti-IGF-IR antibodies, were also disappointing, and this has been attributed to several potential factors, including increased GH release, IGF-II/IR-A signaling, rescue signaling by alternate RTKs and increased IGF-IR nuclear translocation. Recently it was proposed that IGF-IR targeting by antibodies or kinase inhibitors may result in alternative, kinase-independent ERK signaling mediated via recruitment of interacting proteins such as β-arrestins, limiting the effectiveness of these inhibitors (reviewed in [124,125]).

The IGF-Trap offers key advantages over receptor targeting antibodies and small-molecule inhibitors. With high specificity for IGF-I and IGF-II, and poor affinity for insulin, the deleterious effects on the physiological functions of insulin can be minimized. Since the IGF-Trap binds circulating ligands, penetration and diffusion into solid tumors are not major obstacles to efficacy, although uptake at the tumor site, if achieved, could have the added benefit of neutralizing locally produced ligands. Moreover, the high binding affinity of the IGF-Trap for IGF-II should reduce IGF-II bioavailability for IR-A activation, bypassing one of the major resistance mechanisms to IGF-IR targeting drugs. In addition, the potential of anti-IGF-IR antibodies to act as natural agonists and activate alternate IGF-IR signaling can be circumvented with the use of an IGF-Trap [125], and targeting of the ligands rather than a cell surface receptor should minimize non-desirable side effects due to antibody-dependent cellular cytotoxicity (ADCC) that can be mediated by the Fc portion of cell bound antibodies [126]. Finally, our evidence suggests that the IGF-Trap, by reducing ligand bioavailability can target several components of the tumor microenvironment, further enhancing its inhibitory activity on tumor cell growth. Having established the utility of the KIRA for monitoring IGF-Trap efficacy in vivo, our data suggest that it could provide a surrogate marker for response evaluation and a potential tool for patient stratification. Collectively, there is therefore a compelling rationale for transitioning this technology to the clinic for treatment of malignant disease, either alone or in combination with other treatment modalities. 

## Figures and Tables

**Table 1:** Insulin-like growth factor (IGF) targeting strategies: the pre-clinical and clinical experience.

Target	Approach	Advantages	Disadvantages	Reference
IGF- insulin receptor (IR)	Nucleic acid approach	High specificity via mRNA degradation	Toxicity, challenges in drug delivery and uptake Compensatory signaling through IR-A Low translational potential	[35]
	Antibodies	Induce internalization and downregulation of IGF-IR	Adverse effects on glucose metabolism Hyperglycemia activation of IR-A by IGF-II nuclear translocation of IGF-IR Compensatory receptor tyrosine kinase (RTK) signaling	[36,37]
	Bispecific antibodies	Neutralizing two or more targets improved protein stability to oxidative and thermal stress Inhibit compensatory signaling by other RTKs	Steric hindrance large, reduced intra-tumoral penetration	[38,39]
	Tyrosine kinase inhibitors (TKI)	Cross reactivity with IR	Affects metabolic insulin signaling via IR-B hyperglycemia short half-life	[37,40]
IGF- ligands	Antibodies	Block IGF-IR and IR-A activation Low affinity for insulin minimizes adverse effects on glucose metabolism Reduced ligand bioavailability in the serum	Efficacy depends on IGF-IR expression levels Reduced plasma IGF levels may trigger compensatory feedback mechanisms	[41]
	Traps	Block IGF-IR and IR-A activation Low affinity for insulin minimizes adverse effects on glucose metabolism Reduce ligand bioavailability in the serum Fc fusion proteins increase serum half-life	Size may limit diffusion into the tumor site Oligomerization due to disulfide bonds may affect manufacturability Could potentially trigger a compensatory feedback mechanism upon long-term administration	[41,42]
